# Editorial: Sustainable food procurement for healthy diets in public and private canteens

**DOI:** 10.3389/fnut.2026.1834706

**Published:** 2026-05-12

**Authors:** Francisco Javier Comendador, Ana Ilić, Marika Ferrari, Lorenza Mistura

**Affiliations:** 1 Council for Agricultural Research and Economics (CREA), Research Centre for Food and Nutrition, Rome, Italy; 2 Department of Nutrition, Food Quality and Safety, University of Zagreb Faculty of Food Technology and Biotechnology, Zagreb, Croatia

**Keywords:** behavioral interventions, food environment, food waste, healthy diets, institutional catering, school feeding programs, sustainable food procurement

Public and private food procurement systems play an increasingly important role in shaping food environments and influencing dietary behaviors, offering significant opportunities to promote healthier and more sustainable diets. The Research Topic “Sustainable Food Procurement for Healthy Diets in Public and Private Canteens” aimed to collect contributions that advance knowledge on how food procurement strategies, menu design, behavioral interventions, and waste reduction practices can support the transition toward sustainable diets.

The articles included in this Research Topic explore complementary dimensions of sustainable food procurement, ranging from governance and policy frameworks to practical interventions in institutional food services. Together, they highlight the complexity of integrating nutritional, environmental, economic, and social objectives within food procurement systems across diverse geographical and socioeconomic contexts.

A first set of contributions focuses on school food programs as strategic instruments for sustainability transitions. School meals represent a particularly influential policy domain because they affect not only children's nutrition but also broader food system dynamics. As reported by Avallone et al. in a case study from France, school feeding programs are examined as multisectoral policy tools capable of supporting ecological and agricultural transitions while improving the sustainability of collective catering systems. The study shows how integrating health objectives with environmental and social dimensions can reinforce the transformative role of school canteens. Crucially, the presence of an appropriate regulatory framework emerges as a key enabling factor, as it can provide economic support for sustainable agriculture and foster social inclusion within food procurement systems.

The potential of school feeding programmes to promote sustainability is further explored by Swensson and Tartanac in a conceptual paper focusing on low- and middle-income countries (LMICs). This critical analysis highlights key challenges such as defining “sustainable food,” developing measurable procurement criteria, and balancing environmental, social and economic objectives. As current research focuses predominantly on high-income countries, the authors advocate for multidisciplinary studies specific to LMIC contexts. The full transformative effectiveness of school feeding programmes in these settings can only be achieved by systematically integrating sustainability objectives with existing social priorities.

A concrete example of integrating different dimensions of sustainability in the context of low-income countries is provided by Ahern et al., who examine the inclusion of locally produced “usipa” fish powder (*Engraulicrypris sardella*) in school feeding programmes in Malawi. This strategy aims to improve nutrition whilst strengthening local food systems. The study offers a multidimensional analysis of the availability, affordability, quality, and practicality of incorporating this product into such a context, as well as the costs associated with its production. By examining the trade-offs between these dimensions, the research illustrates how local food procurement within institutional supply systems can simultaneously address nutritional deficiencies, support local producers, and contribute to more sustainable supply chains.

Another contribution addressing school feed programs is provided by Lin et al., who investigated the feasibility of incorporating cheese into primary school meals to address insufficient calcium intake associated with the low provision of dairy products in Chinese school menus. The pilot study demonstrates that children respond positively to cheese-based dishes, particularly ready-to-eat and steamed options, indicating that menu innovation can successfully align nutritional improvements with students' taste preferences. Notably, the authors also suggest that designing menus that better reflect children's preferences may help reduce food waste in school canteens, highlighting the close relationship between menu planning, dietary quality, and sustainability outcomes.

Beyond the school setting, the Research Topic also includes contributions examining food waste and resource efficiency in institutional catering systems. A quantitative study conducted in three hospital canteens in north-eastern Italy by Fiori et al. analyses plate waste in order to assess the nutritional and environmental implications of food waste in collective catering. The results show that a measurable share of the food served remains uneaten. Interestingly, the nutrients most frequently wasted were dietary fiber, plant proteins, and vegetal lipids, suggesting that plant-based foods—such as vegetables, side dishes, and legumes—were more likely to be left on trays. The study also estimates the environmental footprint of wasted food, highlighting associated carbon and water impacts. By combining nutritional, environmental, and behavioral analyses—including differences related to age and sex—the study provides valuable insights for developing targeted strategies to reduce food waste, such as flexible portion sizes, menu optimization, and consumer awareness initiatives. These findings align with broader international policy goals, including the United Nations Agenda 2030 and the European Union's Farm to Fork strategy.

Complementing these policy and practice-oriented perspectives, another contribution within this Research Topic by Zidan et al. focuses on behavioral interventions aimed at promoting more sustainable food choices while reducing environmental impact. The authors present the pilot implementation of the *Ta'am Mustadam* intervention among university students from a Faculty of Medicine and Health Sciences in the United Arab Emirates. The study is based on behavioral theory and evaluated using the RE-AIM framework, a model that evaluates five key dimensions (Reach, Effectiveness, Adoption, Implementation, and Maintenance). It provides insights not only into the effectiveness of the intervention but also into how it operates in real-world settings, thereby facilitating the identification of barriers to future improvements. Finally, it identifies both strengths and limitations of the program, providing insights for refining future interventions and improving their potential impact on motivations relating to food choices.

Lastly, a narrative review of the existing literature by Hey et al. explores the role of climate labeling on university menus as a tool for guiding more sustainable food choices. Most climate labels provide information on greenhouse gas emissions associated with menu items, while some also include indicators related to nitrogen or water footprints. The evidence reviewed suggests that climate labeling can effectively reduce the selection of high-emission foods while increasing the choice of lower-impact options among university students. Although the effectiveness of labels does not appear to vary significantly across socioeconomic groups, the intervention tends to have a stronger influence on women and older students. Overall, the review highlights menu labeling as a practical and scalable strategy for supporting sustainable consumption within institutional dining environments.

Collectively, these contributions highlight the central role of food procurement in promoting healthy and sustainable dietary transitions, while emphasizing the need for integrated approaches that balance nutritional, environmental, economic and social objectives as well as address potential trade-offs and synergies among them. From a policy perspective, they underline the importance of supportive regulatory frameworks and coordinated actions to enable the effective implementation of sustainable procurement practices across institutional settings. Future research should prioritize the development of robust, possibly harmonized methodologies, particularly for assessing environmental impacts, food waste, and diet quality, to improve comparability and better capture the multiple impacts of food supply systems. Expanding the geographical scope of the evidence—especially in low- and middle-income countries—will also be essential.

